# The complete chloroplast genome of *Sisymbrium altissimum*

**DOI:** 10.1080/23802359.2021.1967800

**Published:** 2021-08-24

**Authors:** Qinghua Xu, Haifeng Lin

**Affiliations:** a College of Landscape Architecture, Nanjing Forestry University, Nanjing, China; b School of Information Science and Technology, Nanjing Forestry University, Nanjing, China

**Keywords:** *Sisymbrium altissimum*, chloroplast genome, phylogenetic analysis

## Abstract

*Sisymbrium altissimum* (*S. altissimum*), belonging to the family Brassicaceae, can grow in soils of all textures, even sand. Here, we reported the complete chloroplast (cp) genome of *S. altissimum* using Illumina sequencing data. The cp genome exhibited a typical quadripartite cycle of 154,042 bp, composing of a pair of inverted repeats (IRs, 26,260 bp) separated by a large single-copy (LSC, 83,912 bp) region and a small single copy (SSC, 17,610 bp) region. A total of 132 genes (87 protein-coding genes, 37 tRNAs, and eight rRNAs) were annotated in this cp genome. Phylogenetic analysis revealed that *S. altissimum* was closely related to *Sisymbrium irio*.

*Sisymbrium altissimum*, commonly known as tumble mustard, is an annual/biennial herb native to Europe and western Asia. It is widely naturalized throughout most of the world, including all of the North America and the Northwest and Southwest of China. The plant grows in soils of all textures and is the tallest species in the genus *Sisymbrium*. The leaves are spicy enough to make wasabi but can also be mixed into salads and other dishes. The scaffold-level genome (scaffolds: 14,597, N50:46 kb) of *S. altissimum* has been released in 2019, but the cp genome of *S. altissimum* has not been published so far. The chloroplast genomes of plants are highly conserved in sequence and structure, which may provide useful information for further species identification and evolutionary study (Wang et al. 2018). In this study, the complete cp genome of *S. altissimum* was first assembled and characterized herein to provide more information for the further analyses of *Sisymbrium* species.

Leaf sample of *S. altissimum* was collected from Cologne, Germany (50°57′29″ N, 6°51′35″ E), and the voucher specimen was deposited at Max Planck Institute for Plant Breeding Research (Haifeng Lin, haifeng.lin@njfu.edu.cn) under the voucher number ERS2461511. The genomic DNA was extracted from leaves using CTAB DNA extraction protocol (Bubner et al. 2004), and DNA quality was assessed by a NanoDrop spectrophotometer (Thermo Scientific, Carls-bad, CA, USA). The DNA sample was fragmented using a Covaris ultrasonic processor (Covaris, USA) to a size of ∼350 bp, then the fragmented DNA was end repaired, ‘A’-tailed, and ligated with the full-length adaptor for Illumina sequencing with further PCR amplification. Sequencing was carried out on the Illumina Hiseq 2500 platform, and the raw sequencing data were first filtered and trimmed by fastp program (Chen et al. 2018). Then, the clean reads were fed into NOVOPlasty v4.2.1 (Dierckxsens et al. 2017) for assembly with the reference genome sequence of *Sisymbrium irio* (GenBank accession number: NC_037838.1). The assembled genome was annotated using PGA (Qu et al. 2019) and corrected manually using Macvector v18.1 (Bi et al. 2016). The final annotated cp genome of *S. altissimum* was submitted to NCBI GenBank (Accession number: MW207296).

The cp genome of *S. altissimum* displayed a typical quadripartite cp genome structure with a total length of 154,042 bp, including one large single-copy (LSC, 83,912 bp), one small single-copy (SSC, 17,610 bp), and a pair of inverted repeats (IRs, 26,260 bp). The GC content of the whole cp genome was 36.42%, which is higher than that of LSC (34.23%) and SSC (29.31%), but lower than IRs (42.3%). A total of 132 genes were identified in the cp genome of *S. altissimum*, including 87 protein-coding genes, eight ribosomal RNA genes, and 37 transfer RNA genes. Among these, 20 genes were found to contain one intron (*atpF*, *ndhA*, *ndhB* × 2, *petB*, *petD*, *rpl2* × 2, *rpoC1*, *rps12* × 2, *rps16*, *trnA-UGC* × 2, *trnG-GCC*, *trnI-GAU* × 2, *trnK-UUU*, *trnL-UAA*, and *trnV-UAC*), and 2 genes contain two introns (*ycf3* and *clpP*). To investigate the phylogenetic relationships of *S. altissimum*, the complete chloroplast sequences of 19 species were selected to construct the phylogenetic tree, and *Ginkgo biloba* was served as outgroup. All of the conserved cp protein-coding genes (76 genes) were first aligned by Muscle (Edgar 2004), and a maximum likelihood (ML) tree with JTT + G + I + F as the best amino acid substitutions model and 1000 bootstrap replicates was then built using MEGA X (Kumar et al. 2018). All positions containing gaps and missing data were eliminated. The phylogenetic tree strongly supported that *S. altissimum* was closely related to *Sisymbrium irio*, and the clade of *Sisymbrium* was evolutionarily closely to the Cruciferous plants, such as *Arabidopsis thaliana* and *Camelina sativa* (Figure 1). *Sisymbrium altissimum* is widely distributed in hillsides, fields and farmland, which have a negative impact on the yield and quality of wheat and other crops. The complete cp genome will provide an important genome resource for effective control of *S. altissimum*, and for taxonomy study of *Sisymbrium*.

**Figure 1.**
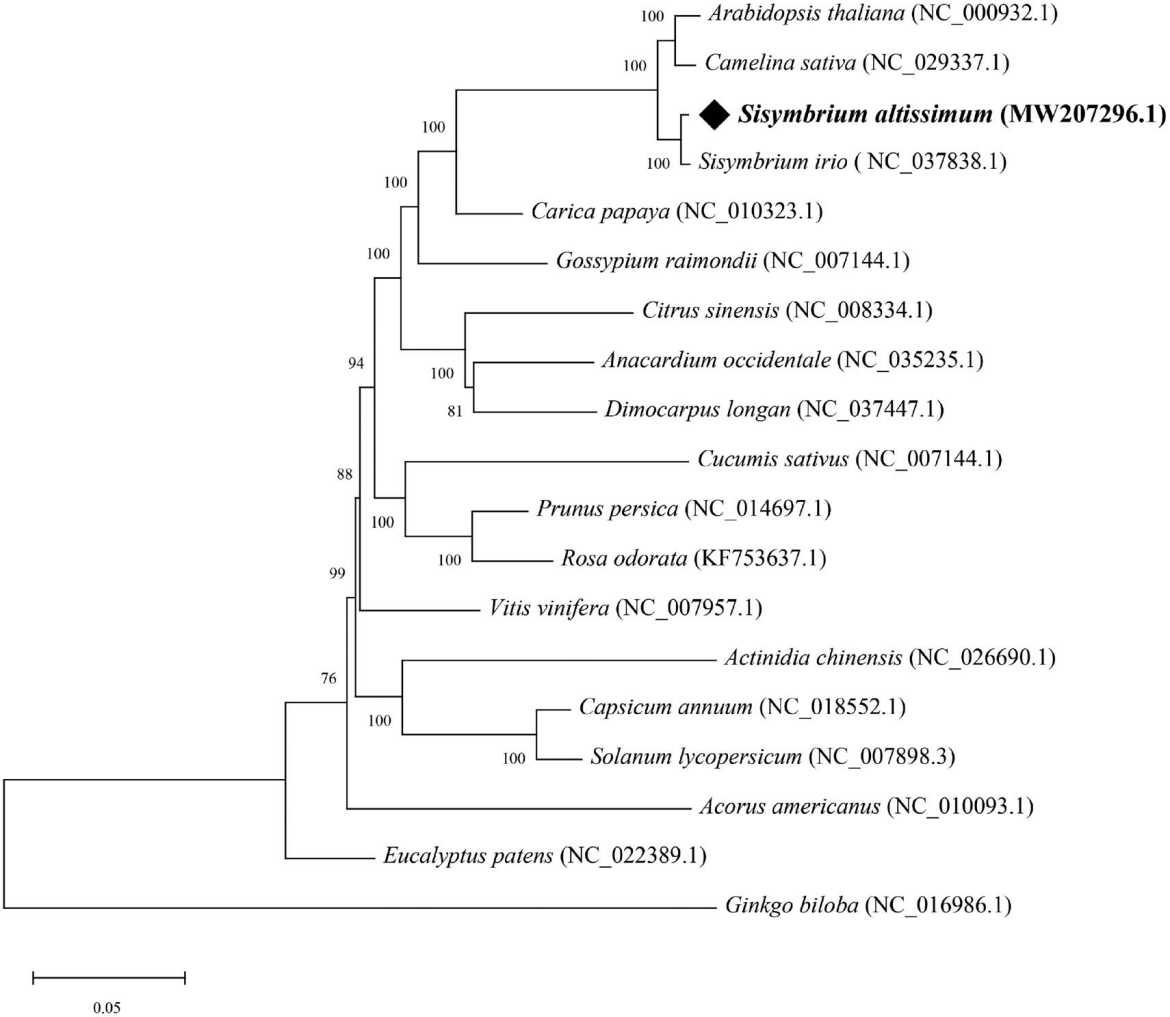
The maximum-likelihood (ML) phylogenetic tree of 19 plant chloroplast genomes based on 76 conserved protein-coding genes. Numbers in the nodes are bootstrap values from 1000 replicates. The GenBank accession numbers for tree reconstruction are listed right to their scientific names.

## Data Availability

The genome sequence data that support the findings of this study are openly available in GenBank of NCBI at https://www.ncbi.nlm.nih.gov/nuccore/MW207296. The associated BioProject, SRA, and Bio-Sample numbers are PRJEB26555, ERR2560543, and SAMEA4640859 respectively.
